# Uneasy bedfellows: Public-Private partnerships for malaria control

**DOI:** 10.5281/zenodo.11046816

**Published:** 2024-04-23

**Authors:** Jacques D. Charlwood

**Affiliations:** 1 Global Health and Tropical Medicine, Instituto de Higiene e Medicina Tropical, Universidade Nova de Lisboa, Rua da Junqueira 100 , 1349-008 Lisbon, Portugal

## Abstract

It is argued that reducing poverty is likely to alleviate malaria transmission and that the way to do this is by reducing inequality. The present capitalist system (as opposed to a straightforward market) tends to erode equality and promote profit over product. This may extend to the manufacture of bednets, bought by agencies rather than individual consumers, whose products may suffer from built in obsolescence. It is argued that better quality nets that can be re-impregnated locally are both desired and required.

Derek Charlwood (aka Mzshensy#1) started his career as a medical entomologist in 1974 as a Research Assistant in the laboratory of the legendary Mick Gillies. By 2012 he had risen to become a Senior Research Assistant working for the Liverpool School of Tropical Medicine and so he is definitely ascending the career ladder. He has worked in numerous malaria endemic countries including Brazil, Papua New Guinea, Tanzania, Cambodia, São Tomé and Príncipe, Mozambique and Eritrea.

Robert Bos, in his recent article [1], seems to be reviving the old distinction between the ‘horizontal’ primary health care (PHC) approach to malaria control and the programme-oriented ‘vertical’ one. This in itself is a revival of the ‘is poverty responsible for malaria?’ or ‘is malaria responsible for poverty?’ argument that echoed in the halls of the World Health Organization (WHO) in the 1950’s [2].

If it is largely the former, as I believe, then attacking poverty at the root would seem to be the most appropriate approach. A survey conducted in 2023 of 9,220 researchers around the world, from a range of scientific and academic disciplines, found that more than 90% agree that ‘fundamental changes to social, political, and economic systems’ are needed [3]. As pointed out by Wilkinson & Pickett in a recent commentary in Nature (March 13th, 2024) ‘The world can no longer afford two things: first, the costs of economic inequality; and second, the rich’ [4]. They point out that over the past two years, the world's super-rich 1% have gained nearly twice as much wealth as the remaining 99% combined and in 2019 they emitted as much carbon dioxide as the poorest two-thirds of humanity. In that time more than five million people were added to those living below the poverty line [5]. ‘Confronting the threat of both climate and ecological breakdown will require us to remake the infrastructure and arrangements of the entire global economy. This is a Herculean task, and our time is short’ [6]. We remain like the man who fell from the top storey of the Empire State building who, as he passed the fifth floor was heard to remark ‘So far, so good’. The urgency of these issues cannot be overstated. We should all be putting a major part of our attention as to how to reduce these inequalities. ‘Confronting profound inequalities in both wealth and power within the global economy is neither optional or a distraction from the challenge at hand’ [6].

A more equal world by itself is likely to lead not only to a reduction in malaria transmission, but to an improvement in the overall health of the planet. Our present economic and financial system, capitalism, concentrates wealth and erodes equality which promotes selfish and non-collaborative behaviours and shapes a range of behaviour patterns, incompatible with sustainability. There has to be another way despite the truth that it is easier to imagine the end of the planet than the end of capitalism.

'Equality is essential for sustainability. The science is clear — people in more-equal societies are more trusting and more likely to protect the environment than are those in unequal, consumer-driven ones’ [4]. It is surely shameful to be a billionaire in this day and age.

Inherent to capitalism is the need for constant exponential growth, by definition unsustainable in a finite world. Capitalism even extends its tentacles into the natural world (a great whale, according to the IMF is worth $2 million). ‘Any two commodities of the same value are reduced to an abstract equality with each other. Their specific sensuous features are thus damagingly ignored, as difference is dominated by identity’ [7]. To paraphrase Oscar Wilde, they know the price of everything but the value of nothing. This directly affects the future of humanity on our planet [8].

Our present economic and financial system, capitalism, concentrates wealth and erodes equality which promotes selfish and non-collaborative behaviours and shapes a range of behaviour patterns, incompatible with sustainability. It also is a requirement of a capitalist enterprise that it makes a profit, and this is where difficulties in ‘public-private’ partnerships can occur.

As the Cobbler sings in Chu Chin Chow:

*‘The stouter I cobble the less I earn,*
*For the soles never crack nor the uppers turn,*
*The better my work the less my pay,*
*But work can only be done in one way’*

In the 1950’s motor-car manufacturers realised that work can, however, be done in other ways, and that they could make more money by producing parts that wore out and needed replacing, than by making a long-lasting product and selling it at a higher price. The practice became known as ‘built-in obsolesence’. This approach has extended to many more products. Are bednets immune?

According to WHO nets should last for three years and the insecticide on them should still be effective against mosquitoes at that time. Recent studies have, however, indicated that many nets, despite apparently being 100 denier, only last half that time or even less [9,10]. Net manufacturers do not appear to be cobblers. ‘Improved net durability needs to be urgently addressed, even if higher quality nets are more expensive’ [11].

As pointed out by Bill Gates in his blog of August 14, 2023 [12]: ‘As temperatures go up and extreme weather events become more common, it will get harder to do things like provide bednets, get rid of malaria-carrying mosquitoes, and offer basic health care in the world’s most vulnerable communities.’

It has long been the contention of the present author that ‘the people of Africa would much rather have, and are prepared to pay for, good-quality non-impregnated bednets, than bad-quality impregnated ones’ [13]. More recently, Okumu [14] has come to the same conclusion. In 1992 I helped distribute nets during the Kilombero Malaria Project in Tanzania. These lasted for such a long time that they were given a nickname: ‘Dereki’ nets (after my name ‘Derek’). Many ‘Deriki’ nets lasted for more than *ten* years.

Surely it should be possible to produce a net that lasts as long as ‘Deriki’ nets? Think of the saving in delivery costs.

Retreating such nets, perhaps with different insecticides, should surely be possible. This was formerly a difficulty task due to a number of reasons. In early trials it was considered that the insecticide was the main agent responsible for any anti-malarial effect. The nets needed to be retreated every six months. This meant that having a net with holes was less important than it might otherwise have been. Because they were hard to obtain, nets in general, in Africa and elsewhere, were generally old and so, full of holes. Thus, when UNICEF bought nets, and insecticide to treat them, to Morogoro, Tanzania, the quality of the net took second place. I purchased two bales of 500 nets with the additional insecticide and took them back to Ifakara, where my two house-girls treated them and established their own business selling them. We devised an automatic bioassay for re-impregnation schedule determination and a scheme for free re-impregnation. Within one or two months of use, the vast majority of these nets were, indeed, full of holes (I tore mine the first time I used it). The insecticide was deemed responsible. Even free offers could not induce any of our UNICEF customers to re-impregnate what remained of their nets. And who can blame them?

Even when people had ‘Deriki’ nets, however, people generally objected to paying for re-impregnation, which originally, was free, and so refused to do so. The same was true in many other places. When I went to The Gambia in 2002, the poster child of the effect of bednets on malaria transmission, I found that retreatment rates were 5%, obviously not enough to affect transmission. Maybe we gave up too early on retreating nets at the local level. A whole PHC system for the care and repair of durable nets (including washing, mending and retreating), perhaps via women’s groups, is required.
